# Supplementary material for: A Cluster-Randomised Crossover Pilot Feasibility Study of a Multicomponent Intervention to Reduce Occupational Sedentary Behaviour in Professional Male Employees
Source: Int J Environ Res Public Health. 2021 Sep 2;18(17):9292. doi: 10.3390/ijerph18179292 (PMC8431104; doi:10.3390/ijerph18179292)
Supplement: Supplementary file 1 [file ijerph-18-09292-s001.zip › ijerph-1336711-supplementary.pdf]

International Journal of
Environmental Research and Public Health

MDPI

Supplementary Table S1

Developed from:

Tong A, Sainsbury P, Craig J. Consolidated criteria for reporting qualitative research (COREQ): a 32-item checklist for interviews and focus groups. *International Journal for Quality in Health Care*. 2007. Volume 19, Number 6: pp. 349 – 357

**No. Item**	**Guide questions/description**	**Reported on Page #**
**Domain 1: Research team and reflexivity**		
Personal Characteristics		
1. Interviewer/facilitator	Which author/s conducted the interview or focus group?	5
2. Credentials	What were the researcher's credentials? E.g. PhD, MD	5
3. Occupation	What was their occupation at the time of the study?	5
4. Gender	Was the researcher male or female?	5
5. Experience and training	What experience or training did the researcher have?	5
Relationship with participants		
6. Relationship established	Was a relationship established prior to study commencement?	5
7. Participant knowledge of the interviewer	What did the participants know about the researcher? e.g. personal goals, reasons for doing the research	4
8. Interviewer characteristics	What characteristics were reported about the inter viewer/facilitator? e.g. Bias, assumptions, reasons and interests in the research topic	4, 5
**Domain 2: study design**		
Theoretical framework		
9. Methodological orientation and Theory	What methodological orientation was stated to underpin the study? e.g. grounded theory, discourse analysis, ethnography, phenomenology, content analysis	6
Participant selection		
10. Sampling	How were participants selected? e.g. purposive, convenience, consecutive, snowball	4
11. Method of approach	How were participants approached? e.g. face-to-face, telephone, mail, email	4
12. Sample size	How many participants were in the study?	8
13. Non-participation	How many people refused to participate or dropped out? Reasons?	8
Setting		

14. Setting of data collection	Where was the data collected? e.g. home, clinic, workplace	3
15. Presence of non-participants	Was anyone else present besides the participants and researchers?	5
16. Description of sample	What are the important characteristics of the sample? e.g. demographic data, date	9
Data collection		
17. Interview guide	Were questions, prompts, guides provided by the authors? Was it pilot tested?	5
18. Repeat interviews	Were repeat inter views carried out? If yes, how many?	N/A
19. Audio/visual recording	Did the research use audio or visual recording to collect the data?	5
20. Field notes	Were field notes made during and/or after the interview or focus group?	5
21. Duration	What was the duration of the inter views or focus group?	5
22. Data saturation	Was data saturation discussed?	5
23. Transcripts returned	Were transcripts returned to participants for comment and/or correction?	5
**Domain 3: analysis and findings**		
Data analysis		
24. Number of data coders	How many data coders coded the data?	6
25. Description of the coding tree	Did authors provide a description of the coding tree?	N/A
26. Derivation of themes	Were themes identified in advance or derived from the data?	6
27. Software	What software, if applicable, was used to manage the data?	6
28. Participant checking	Did participants provide feedback on the findings?	6
Reporting		
29. Quotations presented	Were participant quotations presented to illustrate the themes/findings? Was each quotation identified? e.g. participant number	9-12
30. Data and findings consistent	Was there consistency between the data presented and the findings?	9-12
31. Clarity of major themes	Were major themes clearly presented in the findings?	9-12
32. Clarity of minor themes	Is there a description of diverse cases or discussion of minor themes?	9-12

TIDieR
Template for Intervention Description and Replication

The TIDieR (Template for Intervention Description and Replication) Checklist*:

Information to include when describing an intervention and the location of the information

Item number	Item	Where located **	
		Primary paper (page or appendix number)	Other † (details)
	BRIEF NAME		
1.	Provide the name or a phrase that describes the intervention.	____3_______	____________
	WHY		
2.	Describe any rationale, theory, or goal of the elements essential to the intervention.	____1_______	____________
	WHAT		
3.	Materials: Describe any physical or informational materials used in the intervention, including those provided to participants or used in intervention delivery or in training of intervention providers. Provide information on where the materials can be accessed (e.g. online appendix, URL).	online appendix	____________
4.	Procedures: Describe each of the procedures, activities, and/or processes used in the intervention, including any enabling or support activities.	3-5	____________
	WHO PROVIDED		

5.	For each category of intervention provider (e.g. psychologist, nursing assistant), describe their expertise, background and any specific training given.	5	____________
	HOW		
6.	Describe the modes of delivery (e.g. face-to-face or by some other mechanism, such as internet or telephone) of the intervention and whether it was provided individually or in a group.	4-5	____________
	WHERE		
7.	Describe the type(s) of location(s) where the intervention occurred, including any necessary infrastructure or relevant features.	3	____________
	WHEN and HOW MUCH		
8.	Describe the number of times the intervention was delivered and over what period of time including the number of sessions, their schedule, and their duration, intensity or dose.	3-5	____________
	TAILORING		
9.	If the intervention was planned to be personalised, titrated or adapted, then describe what, why, when, and how.	na	____________
	MODIFICATIONS		
10.‡	If the intervention was modified during the course of the study, describe the changes (what, why, when, and how).	na	____________
	HOW WELL		
11.	Planned: If intervention adherence or fidelity was assessed, describe how and by whom, and if any strategies were used to maintain or improve fidelity, describe them.	7	____________

12.‡	Actual: If intervention adherence or fidelity was assessed, describe the extent to which the intervention was delivered as planned.	na	__________

** Authors - use N/A if an item is not applicable for the intervention being described. Reviewers – use '?' if information about the element is not reported/not sufficiently reported.

† If the information is not provided in the primary paper, give details of where this information is available. This may include locations such as a published protocol or other published papers (provide citation details) or a website (provide the URL).

‡ If completing the TIDieR checklist for a protocol, these items are not relevant to the protocol and cannot be described until the study is complete.

* We strongly recommend using this checklist in conjunction with the TIDieR guide (see *BMJ* 2014;348:g1687) which contains an explanation and elaboration for each item.

* The focus of TIDieR is on reporting details of the intervention elements (and where relevant, comparison elements) of a study. Other elements and methodological features of studies are covered by other reporting statements and checklists and have not been duplicated as part of the TIDieR checklist. When a **randomised trial** is being reported, the TIDieR checklist should be used in conjunction with the CONSORT statement (see www.consort-statement.org) as an extension of **Item 5 of the CONSORT 2010 Statement.** When a **clinical trial protocol** is being reported, the TIDieR checklist should be used in conjunction with the SPIRIT statement as an extension of **Item 11 of the SPIRIT 2013 Statement** (see www.spirit-statement.org). For alternate study designs, TIDieR can be used in conjunction with the appropriate checklist for that study design (see www.equator-network.org).
